# A generic method to synthesise graphitic carbon coated nanoparticles in large scale and their derivative polymer nanocomposites

**DOI:** 10.1038/s41598-017-12200-1

**Published:** 2017-09-19

**Authors:** Nannan Wang, Zhuxian Yang, Fang Xu, Kunyapat Thummavichai, Hongmei Chen, Yongde Xia, Yanqiu Zhu

**Affiliations:** 1 College of Engineering, Mathematics and Physical Sciences, University of Exeter, Exeter, EX4 4QF United Kingdom; 2 Faculty of Engineering, the University of Nottingham, Nottingham, NG7 2RD United Kingdom; 3 School of Materials Science and Engineering, Jiangsu University of Science and Technology, No. 2 Mengxi Road, Zhenjiang, 212003 China

## Abstract

A versatile Rotary Chemical Vapour Deposition (RCVD) technique for the *in-situ* synthesis of large scale carbon-coated non-magnetic metal oxide nanoparticles (NPs) is presented, and a controllable coating thickness varying between 1–5 nm has been achieved. The technique has significantly up-scaled the traditional chemical vapour deposition (CVD) production for NPs from mg level to 10 s of grams per batch, with the potential for continuous manufacturing. The resulting smooth and uniform C-coatings sheathing the inner core metal oxide NPs are made of well-crystallised graphitic layers, as confirmed by electron microscopy imaging, electron dispersive spectrum elemental line scan, X-ray powder diffractions and Raman spectroscopy. Using nylon 12 as an example matrix, we further demonstrate that the inclusion of C-coated composite NPs into the matrix improves the thermal conductivity, from 0.205 W∙m^−1^∙K^−1^ for neat nylon 12 to 0.305 W∙m^−1^∙K^−1^ for a 4 wt% C-coated ZnO composite, in addition to a 27% improvement in tensile strength at 2 wt% addition.

## Introduction

As one of the most abundant elements in the world, carbon has accompanied with human beings from the ancient time as the main energy source to the 21^st^ century, and now becomes one of the most studied materials in laboratories. Its several different forms, such as graphite, diamond, fullerenes, carbon nanotubes and graphene, have all attracted immense research interests owing to their excellent thermal, mechanical, optical and electrical properties^1–6^, and to the great potentials for key engineering applications in the fields of solid lubricants, supercapacitors and battery electrodes, nanodevices and composites. Metal oxides are widely used in catalysis, photoluminescence, composites, thermal-resisting, semiconducting industries, and in surface protection from acid and alkali corrosions, due to their super hardness and richness in material choices and properties, and highly thermal and chemically stable features^7–10^. The joining of carbon and metal oxides could offer combined advantages of both materials in terms of their chemical and physical properties, and investigation into such combinations in the bulk forms had been conducted previously and indeed extended their applications in sorbents, batteries and supercapacitors, etc^3,5,11^.

At nanoscale, new properties originating from the nanoscale effect will make the joining of carbon and metal oxides more interesting^12^, and significant properties are expected. With the previous success in metal oxide NPs production, C-coating on NPs to form core-shell structured composite NPs is logically an ideal way to joining them together. Given the vast amounts of literature available for surface modification of carbon nanomaterials^13–16^, carbon sheathed NPs could adopt the layered hexagonal graphitic structure of carbon, offering high conductivity, flexibility and versatility towards applications. Indeed, early attempts had prepared such carbon-wrapped metal oxide composites using plasma sputtering deposition, CVD, solvothermal synthesis and template growth^17–20^, among which the CVD method allowed for easy thickness control and tuneable layer quality^21–23^. However, a normal static CVD failed to scale-up, and coating individual particles was hard to achieve due to the sintering effect and strong tendency of NP agglomeration^24^. A recently improved CVD system was reported by Liu *et al*., and they achieved a small amount of high quality onion-like layered graphitic carbon coating on CoO, named as CoO@C^25^, which exhibited a very good cyclic stability in lithium ion batteries. To tackle the key problem of agglomeration and realise a truly large scale production, Xu *et al*. have developed a rotary chemical vapour deposition (RCVD) system^26^, and have successfully realised an efficient, effective and continuous production of C-wrapped inorganic fullerene WS_2_ composite NPs, IF-WS_2_@C. The physical rotary movement of the RCVD effectively counters the NP agglomeration, whilst the CVD part maintains the simple, uniform, and controllable advantages of a conventional static reactor for the synthesis of IF-WS_2_@C composite NPs.

In this study, we will investigate the full potential of the RCVD process, extend and explore the C-coating in a wide range of interesting micro- or nano-sized non-magnetic metal oxides, including MO_X_ (M = Ce, Cr, Zr, Y, Ti and Zn, where x = 1, 1.5 and 2). We then further demonstrate the actual advantages of the resulting MO_X_@C composite NPs in example polymeric composites, against the un-coated NPs. Nylon 12 has been chosen for this study because of its superb ability of loading reinforcing materials, with many examples for comparison, including the hotspot carbon materials such as carbon nanotubes and graphene^27,28^.

## Experimental

All the metal oxide particles (<100 nm) and styrene (analytical reagent 99.99%) were purchased from Sigma-Aldrich. Ethanol and acetone (analytical reagent 99.99%) were bought from Fisher-Scientific.

### Preparation of graphitic carbon coated metal oxide nanoparticles (MO_X_@C NPs)

**Figure 1:**
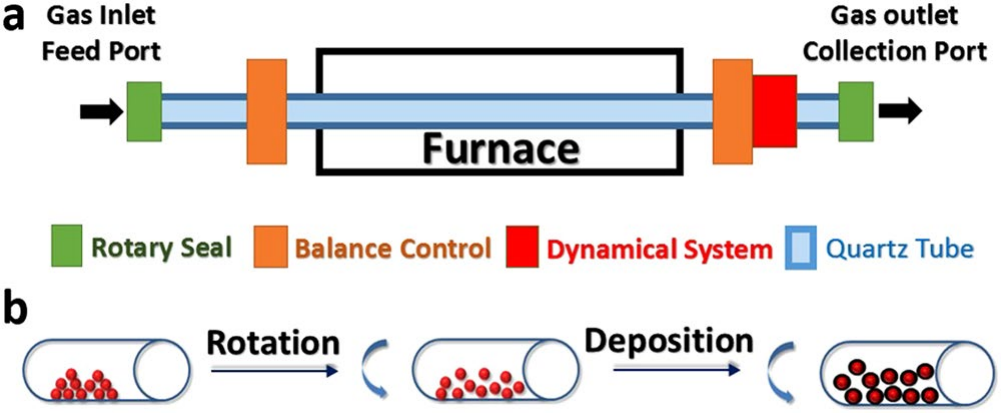
A sketch of (**a**) the Rotary Chemical Vapour Deposition (RCVD) system, and (**b**) the carbon coating processes during the RCVD synthesis.

Various C-coated metal oxides were prepared following the modified RCVD method reported by Xu *et al*.^29^. As illustrated in Fig. 1a, the raw material was sealed in the quartz tube and protected by argon flow. In addition, a mechanical rotation was applied to the quartz working tube and the particles would be shaken and separated by the friction force from the tube wall and its gravity which anti-countered the self-agglomeration and avoided sintering. Typically, 10 g of different metal oxide NPs were dispersed respectively into 100 ml ethanol under vigorous magnetic stirring for 1 h without any surfactant, followed by ultrasonic probe dispersion for 30 min. The resulting mixture suspension was first dried at 80 °C in a long tube furnace (VecStar Ltd.) to obtain dry powder, which was then ground in an agate mortar to eliminate the bulk residual, and finally transferred into the inlet part of the long quartz tube (2 meters long with an inner diameter of 35 mm). The carbon precursor was prepared by ultrasonic mixing of acetone and styrene solutions with a volume ratio of 4:1. The reaction zone of the system was sealed by the designed rotary seals at both ends. The rotation system was started with Ar flow of 100 ml/min, and 2 ml/h of the carbon precursor was introduced into the reaction zone for 15 to 60 min, at temperatures varying from 550 °C to 1000 °C, and then naturally cooled down to room temperature under Ar protection. After the RCVD process, a controllable layer of carbon, depending on the reaction duration and temperature, precursor concentration and flow rate, etc., was deposited, as illustrated in Fig. 1b.

### Preparation of MO_X_@C-Nylon 12 nanocomposite

A 1:1 mixture of ethanol and distilled water was used to disperse the powders. Taking ZnO@C composite NPs as an example, the fraction of ZnO@C NPs in the nylon 12 matrix powder is 1, 2, and 4 wt%, respectively. First, the ZnO@C NPs and nylon 12 powder were separately dispersed in 100 ml of the alcohol-water solution, and then followed by 1 h vigorous magnetic stirring. The two resulting suspensions were mixed together and subjected to powerful ultrasonic-probe treatments for 15 min. The well-mixed suspension was heated at 100 °C under magnetic stirring for 1 h on a hot plate to remove most of the solvent, followed by drying in an oven at 120~140 °C for 24~48 h for a complete removal of the solvent. The mixed powders were transferred onto a glass slide of 190 to 210 °C sitting on a hot plate. After a few minutes, the powders were melted completely to form a thin film (*m.p*. of nylon 12, 178~180 °C^30^), and the glass slide was then removed from the hot plate and allowed to cool naturally. The resulting composite film could be easily peeled off from the glass slide for subsequent study.

### Characterisations

X-ray diffraction (XRD) patterns were acquired using a Bruker D8 Advance diffractometer working with a Cu *Kα* (Ni-filtered) radiation at λ = 0.15418 nm. The testing 2θ angles were 15–40°, with a scanning step size of 2θ = 0.03°. The Raman spectra were obtained from a Renishaw Raman spectroscope with a laser wavelength of 532 nm, for the evaluation of the carbon vibration modes of the MO_X_@C NPs. Thermogravimetric analyses (TGA) were carried out with a TA instrument - SDT Q600D machine, in order to assess the thermal stability of the composites, at a heating rate of 10 °C∙min^−1^ under air flow of 100 cm^3^∙min^−1^. Measurements were recorded from room temperature to 700 °C, using an average sample mass of 10 mg. The morphology analysis of the MO_X_@C NPs were studied by Scanning Electron Microscope (HITACHI S3200N SEM). In order to increase the contract for those inorganic particles, all the samples were subjected to gold pre-coated treatment using plasma sputtering deposition. Transmission Electron Microscope (TEM) images were acquired from a JEM-2100 TEM attached with an energy dispersive spectrum (EDS–Oxford EDS instrument) operated at 200 kV. The scanning transmission mode was used to evaluate the distribution and structure of the coated carbon layers. UV-Vis spectrum (JENWAY-6715 UV-Vis Spectrophotometer) was used to measure the absorbance intensity of the MO_X_@C NPs suspension with a concentration of 5 mg∙ml^−1^. The measurement step was 0.1 nm and the testing wavelength ranging from 200 to 600 nm. NETZSCH LFA 467 HypeFlash machine was used to analyse the thermal conductivities of the MOx@C-nylon 12 nanocomposites, with a specimen size of 10 × 10 mm and thickness of *ca*. 0.5 mm. The tensile property was measured using a mechanical testing machine (Lloyd Instruments), with a specimen size of 50 × 5 × 0.5 mm and a gauge length of 30 mm at a pulling rate of 5 mm∙min^−1^. At least 5 samples were tested for each batch of composites.

## Results and Discussion

**Figure 2:**
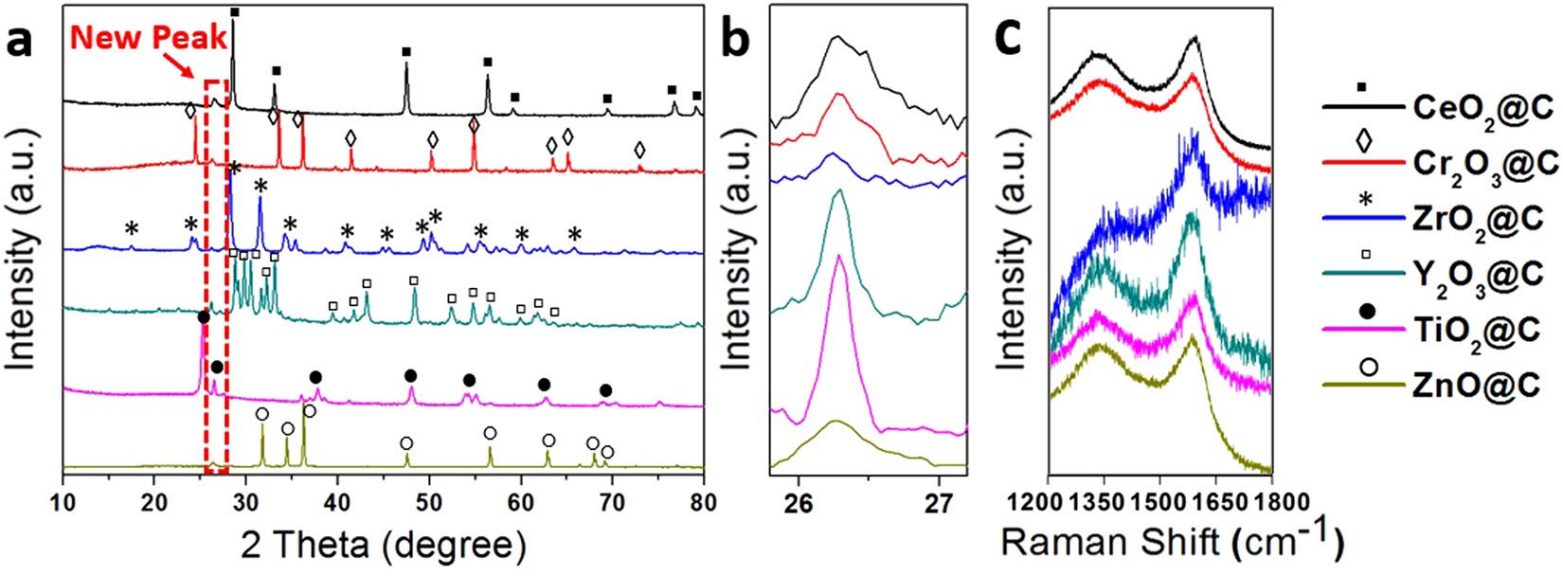
(**a**) and (**b**) XRD patterns and (**c**) Raman spectra of the MO_X_@C NPs.

All the MO_X_@C NPs were synthesised under the same conditions of 30 min at 800 °C. The structures of the MO_X_@C NPs were characterized by XRD, and the results are shown in Fig. 2a. Compared with the XRD patterns of the original metal oxides, a new peak at 2θ around 26.2° for all MO_X_@C NP samples is observed. Different from the amorphous carbon showing a broad peak at 2θ around 26.2°, this weak but sharp peak is believed to originate from the (002) diffraction of graphitic carbon^31^, indicating a high level of graphitisation for the coating layer. The enlarged peaks from 26° to 27° are shown in Fig. 2b. In the case of TiO_2_, its (110) peak around 26° overlaps with the (002) peak of graphitic carbon^32^, which results in the sharpest diffraction peak with highest intensity around 26°. The intensity ratio of carbon to metal oxide is quite low, implying that the amount of the carbon in the MO_X_@C NPs is small therefore the coating should be thin.

The Raman spectra of the NPs are presented in Fig. 2c. All MO_X_@C NPs show peaks around 1350 cm^−1^ (D band) and 1590 cm^−1^ (G band) those are originated from the bond stretching of pairs of sp^2^ atoms in both chains and rings and sp^2^ atoms in the breathing vibration modes in rings, respectively^33,34^. The D band arises from the disordered carbon, and the G band represents the sp^2^ – hybridized crystalline carbon. Therefore, the intensity ratio of D band to G band indicates the crystallization level of the carbon coating, i.e. the higher value of I_D_/I_G_, the more defects inside the specimen^33^. In these MO_X_@C NPs, the ZrO_2_@C NPs exhibit the lowest I_D_/I_G_ value therefore it possesses the highest graphitic crystallization level^35^, and the second is the Y_2_O_3_@C NPs, whereas the rest of MO_X_@C NPs all show fairly good crystallization.

**Figure 3:**
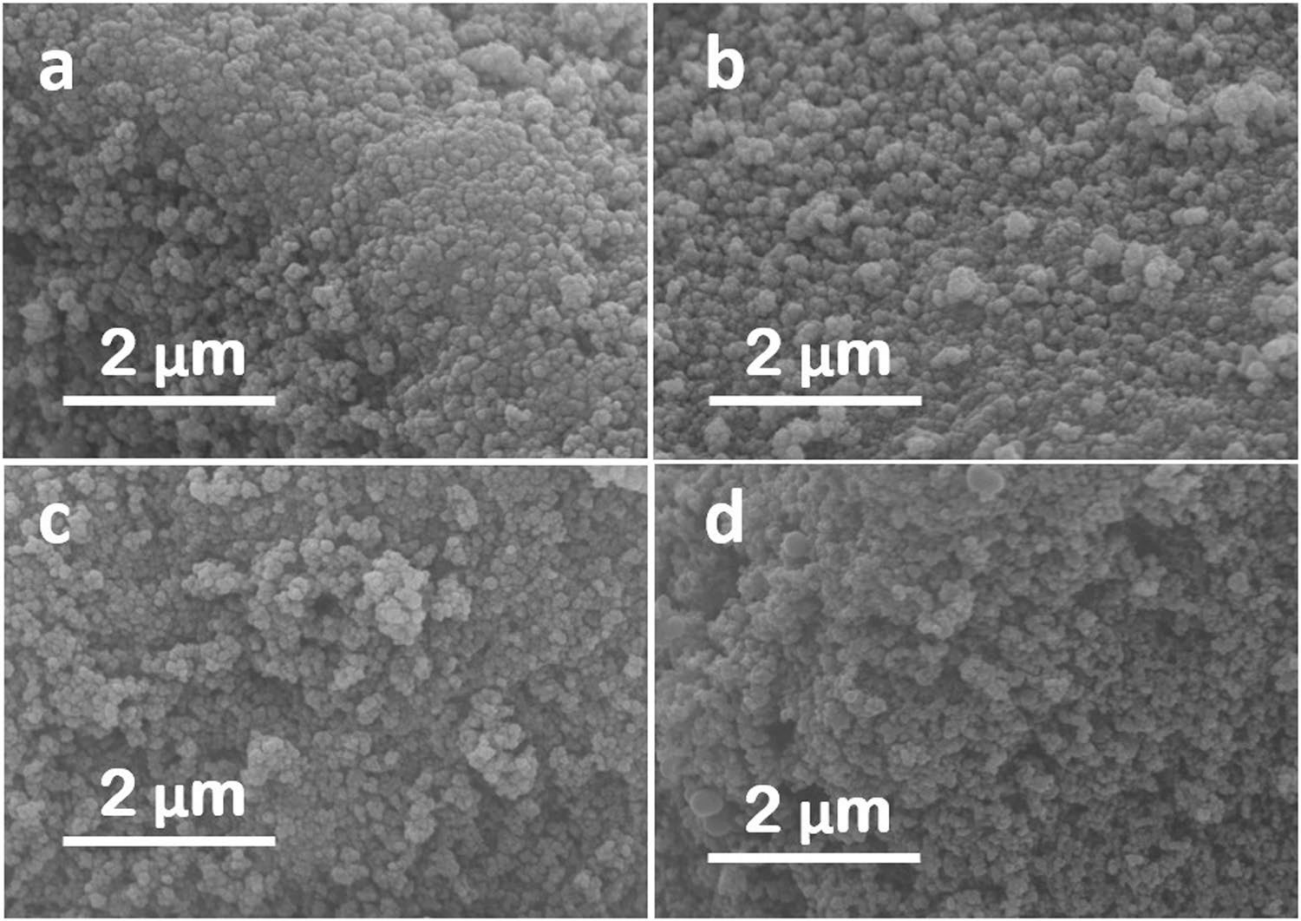
Representative SEM images of selected NPs: (**a**) ZnO and (**b**) ZnO@C, and (**c**) TiO_2_ and (**d**) TiO_2_@C.

The particle morphologies before and after the C-coating were examined by SEM to visualise the overall feature of the NPs, and example results of ZnO and TiO_2_ are shown in Fig. 3. It is obvious that both types of NPs exhibit similar particle size distribution, with/without the C-coating. There were no visible morphological differences under SEM examination for all NPs, which suggest that the RCVD appears to be an effective and versatile way for the production of a wide range of C-coated metal oxide NPs, without changing their original morphologies and free of agglomeration.

**Figure 4:**
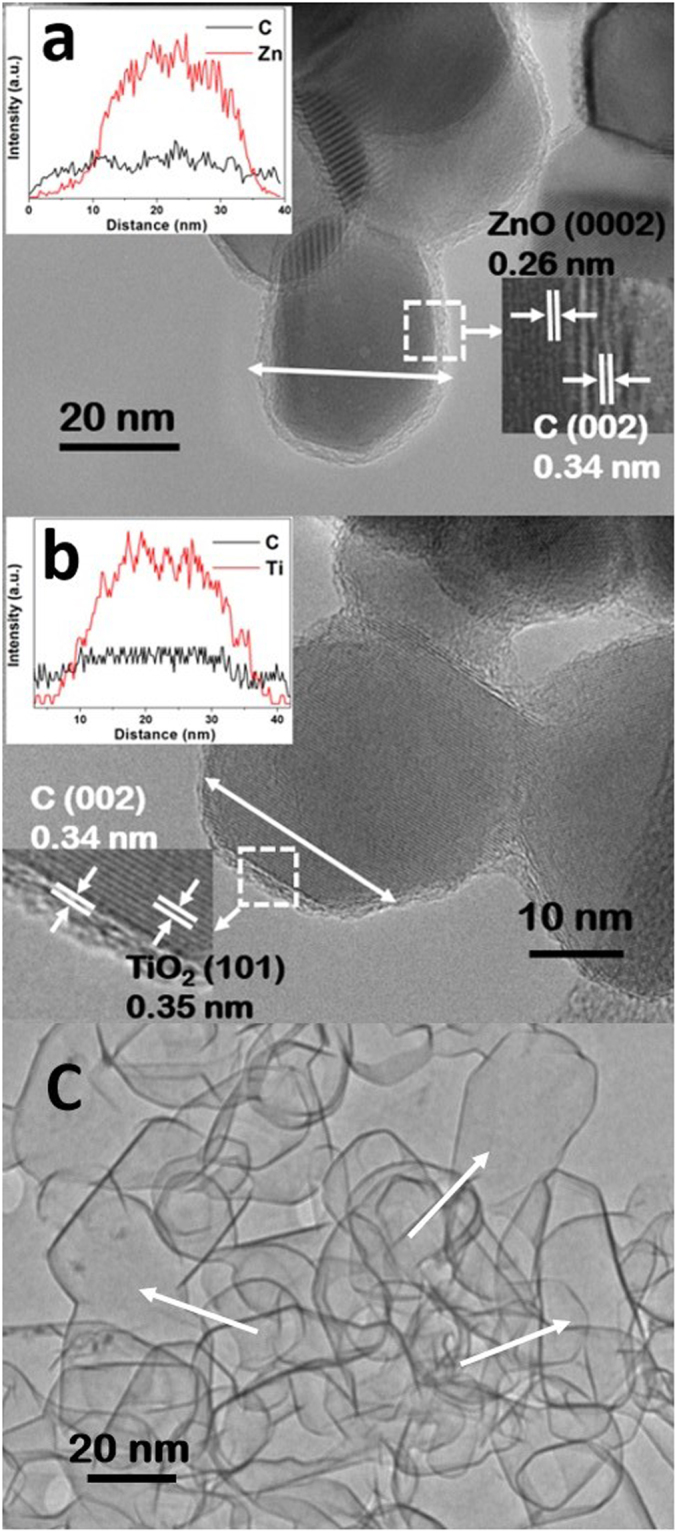
TEM images of (**a**) ZnO@C and (**b**) TiO_2_@C NPs. The insets are the corresponding EDS-line scan patterns; and (**c**) the carbon shell after the removal of ZnO core.

The detailed structure and morphology features of individual MO_X_@C NPs were further investigated by TEM, and the resulting images are shown in Fig. 4. In Fig. 4a, the ZnO@C NPs exhibit an average particle size of *c.a*. 30–50 nm, with a bright carbon shell homogenously covering the dark ZnO core. The zoomed image of the selected area shows the thickness of the coating < 2 nm with a lattice fringe of ~0.34 nm which corresponds to the (002) graphitic carbon^31^. This observed very thin coating is in consistent with the weak (002) peak intensity obtained in above XRD analyses. The EDS line scan clearly demonstrates that the C-coating is smoothly sheathed the entire particle surface. In particular, the almost constant C intensity signals when crossing the particle confirm that the coating has a uniform thickness. The TiO_2_@C NPs exhibit similar morphology and structures to the ZnO@C NPs, as shown in Fig. 4b.

We further demonstrated the uniform feature of the carbon shell by completely removing the ZnO core. This was conducted by re-heating the ZnO@C NPs at 800 °C under H_2_ to first reduce the ZnO to metal Zn, which then evaporated leaving behind the carbon shells. The resulting shells are displayed in Fig. 4c. The majority of the empty shells exhibit an individual, complete and spherical characteristic that resembles the size and shape of the original ZnO NPs, whilst a few shells show an irregular, large feature. These large shells must have formed from two or three adjacent particles that were wrapped together (arrowed in Fig. 4c). It is noteworthy that although not all the NPs are sheathed individually during the RCVD, nevertheless the overall quality of the coating is very high.

As presented above, all the metal oxide particles are in nanoscale, which makes them prone to agglomeration at high temperature, leading to the formation of bulk coating containing a number of particles. This is one of the challenges for conventional techniques to scale-up the production of NPs beyond laboratory level. In this regard, the novel RCVDs can effectively prevent the particle agglomeration while generating individual MO_X_@C NPs in large scale without damaging the original structure of the metal oxide particles. The constant moving of NPs during the RCVD process, as shown in Fig. 1b, effectively allows the atomic carbons to make contact with the entire NP surface (in contrast to a static CVD where part of the stationary NP surface will not be exposed to the carbon atoms) so that a fully covered surface with a uniform thickness can be achieved. Furthermore, there are no isolated amorphous carbon spheres found as a by-product, which verifies that the chosen RCVD parameters are appropriate for the generation of graphitic coating.

**Figure 5:**
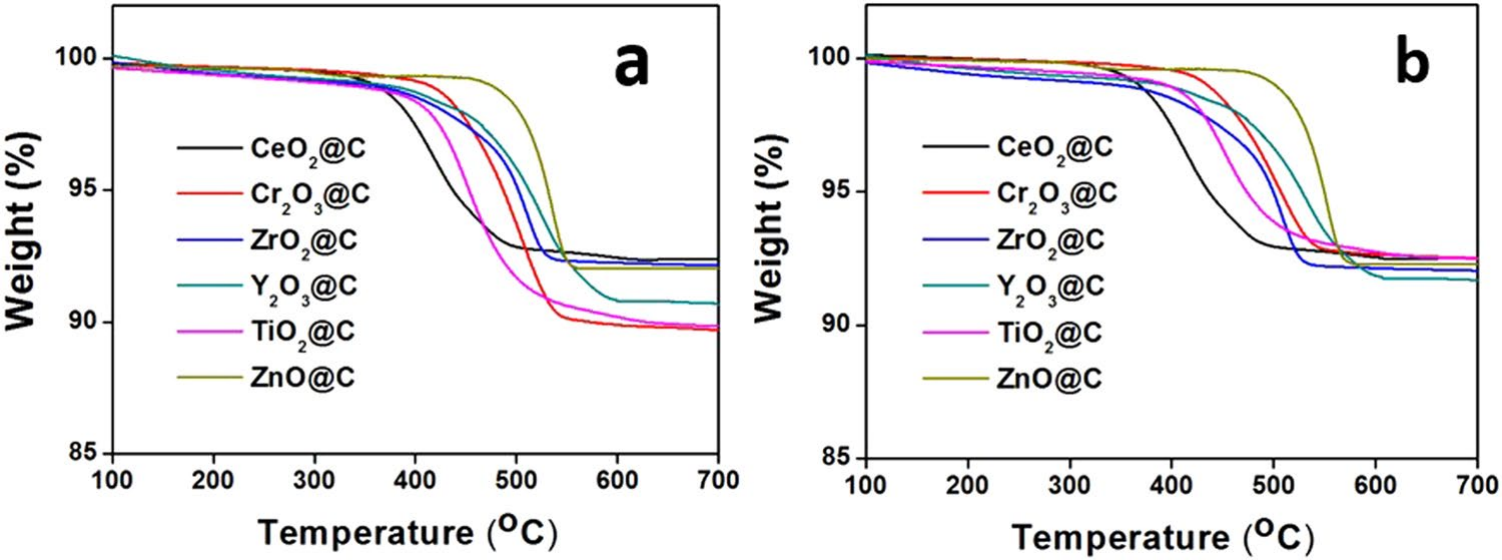
(**a**) The original and (**b**) normalised TGA curves of different MO_X_@C NP specimens.

The thermal stability of various MO_X_@C NPs is shown in Fig. 5a, and the degradation is due to the oxidation or burning of the outer carbon shell to form CO_2_. These profiles show different starting and ending degradation temperatures, and different degradation speeds which are a reflection of the graphitisation level, as highly graphitised carbon will be oxidised slowly at higher temperature^36^. The ZnO@C NPs show the highest starting degradation temperature (T_S_) at 463 °C, while the CeO_2_@C NPs have the lowest T_S_ at 361 °C, and the other NPs are around 390–400 °C. The ending degradation temperatures (T_E_) for these MO_X_@C NPs are between 550–570 °C, except CeO_2_@C NPs which is at 500 °C. Accordingly, the slopes of weight loss are quite complex, associated with the onset T_S._ These weight loss characteristics are comparable to or even better than many other forms of nanostructured carbon materials, such as graphene and carbon nanotubes as coating layers demonstrating the high quality of graphitic coating^37,38^.

From the Raman spectra, the ZrO_2_@C NPs have relatively better crystallisation with the lowest I_D_/I_G_ (=0.58) than the other NPs (Fig. 2c). However, their T_E_ does not appear to be the highest, only sitting in the middle, and is comparable to that of Cr_2_O_3_@C and TiO_2_@C NPs. Also for the ZnO@C NPs, although their XRD profile shows the broadest (002) peak and their Raman spectrum has higher I_D_/I_G_ value (=0.96), both suggesting relatively poorer crystallised coating than others, they exhibit the highest T_E_. Therefore, the quality of the carbon coating is not only associated with the crystallization level, but also influenced by other properties of the core material. The interfaces of each metal oxide are fairly different, hence they could affect both the T_S_ and T_E_ of MO_X_@C NPs. We believe that the different thermal stabilities among these coated NPs should be associated with the individual surface chemistry and crystalline structures of the core, and a better match with those of the coating would lead to higher quality of graphitization and stronger adhesion with the core, resulting in high T_E_. In the case of ZnO NPs, it has a lattice constant of 0.325 nm^39^, which matches very well with the 0.34 nm layer spacing observed in the coating, which promotes the coating growth and the formation of a strong adhesion to the ZnO core. Therefore, the ZnO@C NPs exhibit the highest T_E_.

To contrast the coating amounts against different metal oxides is quite complicated, because the NPs have different densities (i.e. different numbers of NPs for 10 mg samples used for TGA), therefore the percentage of weight loss does not necessarily to be a direct reflection of the carbon weigh loss for one particle. By assuming a uniform particle size and particle stacking density, we can approximately normalise the percentage of the weight loss shown in Fig. 5a, and have obtained Fig. 5b for different MO_X_@C specimens by using the ZnO NPs as a standard. The normalised results have shown that the residual weights for all MO_X_@C NPs are *ca*. 92%. Therefore, the C-coating on different NPs is nearly constant. This analysis offers further evidence that the RCVD process can precisely control the carbon coating thickness, to produce MO_X_@C NPs at large scale.

**Figure 6:**
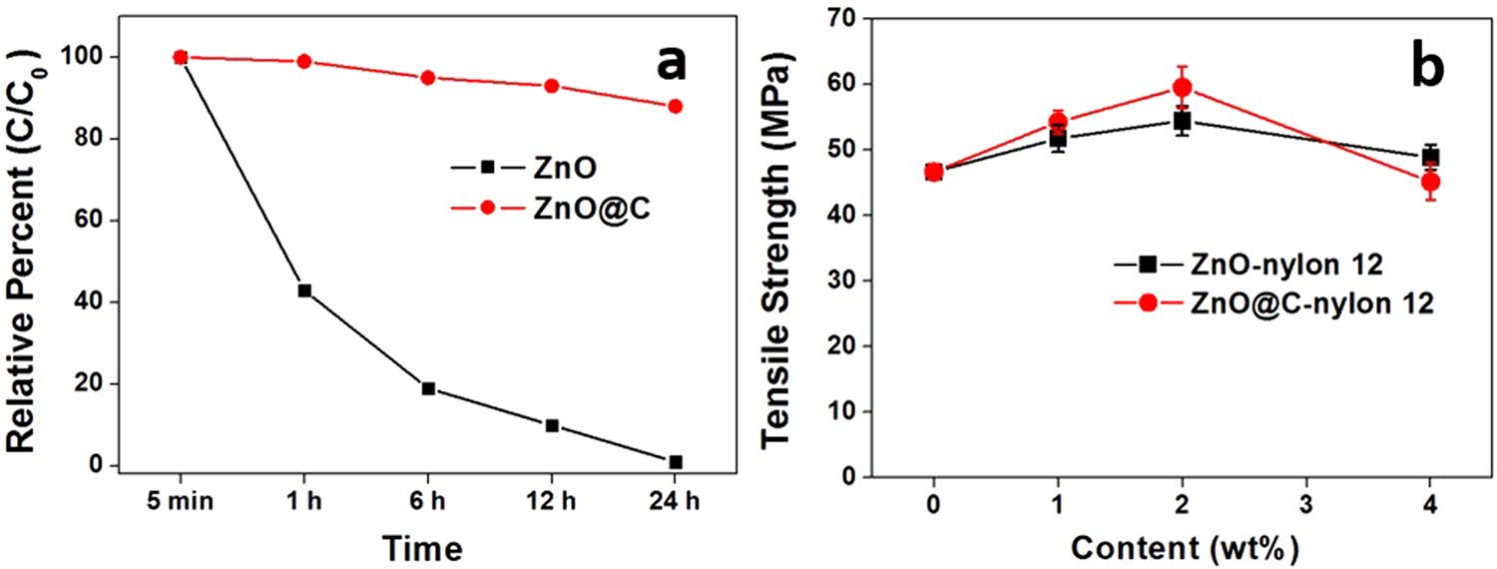
(**a**) UV-Vis absorbance spectra intensity comparison of the plain ZnO and ZnO@C NPs; (**b**) The ultimate tensile strength of the ZnO-nylon 12 and ZnO@C-nylon 12 nanocomposites at different filler contents.

The dispersion property of the NPs in water based solutions was analysed using UV-Vis spectra, as shown in Fig. 6a, to provide an understanding of the key dispersion parameter for future composite fabrication. Using ZnO as a typical example, we prepared the water based suspensions of ZnO and ZnO@C NPs with the assistance of 1 wt% of SDS (Sodium Dodecyl Sulfate), in order to evaluate the coating influence on the sediment rate (opposite to dispersion) of these insoluble NPs. Higher adsorption is an indicator of uniform dispersion, whereas a lower adsorption indicates rapid sediment resulted from agglomeration. A strong ZnO UV-Vis peak at 325 nm was chosen in this study. After 1 h, the absorbance intensity of ZnO@C NPs remained as high as 98%, whilst the plain ZnO only maintained 42% of its original intensity. After 24 h, the plain ZnO NPs was completely dropped to the bottom of the container and there was hardly any adsorption in the spectrum, however the ZnO@C NPs suspension remained over 85% of its original intensity. This result has clearly demonstrated that the surface modified MO_X_@C NPs have significantly improved their dispersal ability with suitable surfactant. The C-coating on the NPs has offered important potentials for anti-countering the agglomeration issues associated with NPs, with a similarly modifiable behaviour to those of the well-documented carbon nanotubes^40^, so that mature surface modification strategies are readily available for achieving strong interface bonding and good dispersions. This excellent surface tuneable feature of the C-coating offered by the RCVD process could open up new opportunities for the fabrication of nanocomposites.

To further validate the above potentials, we attempted to embed selected MO_X_@C NPs into nylon 12, in an effort to take advantages of the thermal and mechanical properties of C-coatings and the intrinsic high hardness features of the metal oxides. By using the simple water/ethanol solvent mixing method, we fabricated ZnO@C NPs-reinforced nylon 12 model composites, in comparison to the plain ZnO NPs-reinforced samples. We will focus our investigation mainly on the evaluation of the key mechanical and thermal properties of the composites, to validate the role of the C-coating in the composites, and further to consolidate the success of the RCVD process.

Further validation was achieved in the tensile strength between the ZnO-nylon 12 and ZnO@C-nylon 12 nanocomposites at filler contents from 1 to 4 wt%, and the results are presented in Fig. 6b. Compared with the plain nylon 12 sample, the ultimate tensile strength of the 2 wt% composites increases to 54.5 MPa and 59.6 MPa, which corresponds to an improvement of 16% and 27% for ZnO and ZnO@C, respectively. By modifying the surface chemistry of the ZnO with the carbon coating, it can not only affect the nanoparticles dispersability in the solution and finally in the composites, but also improve the interface adhesion with the nylon 12 matrix. At 4 wt% however, the ZnO@C-nylon 12 sample exhibits lower ultimate tensile strength than the ZnO. It is well-documented that the enhancing effect of spherical NPs is not that obvious^41^, therefore the 27% improvement at an optimal 2 wt% of Zn@C NPs addition is very impressive.

**Figure 7:**
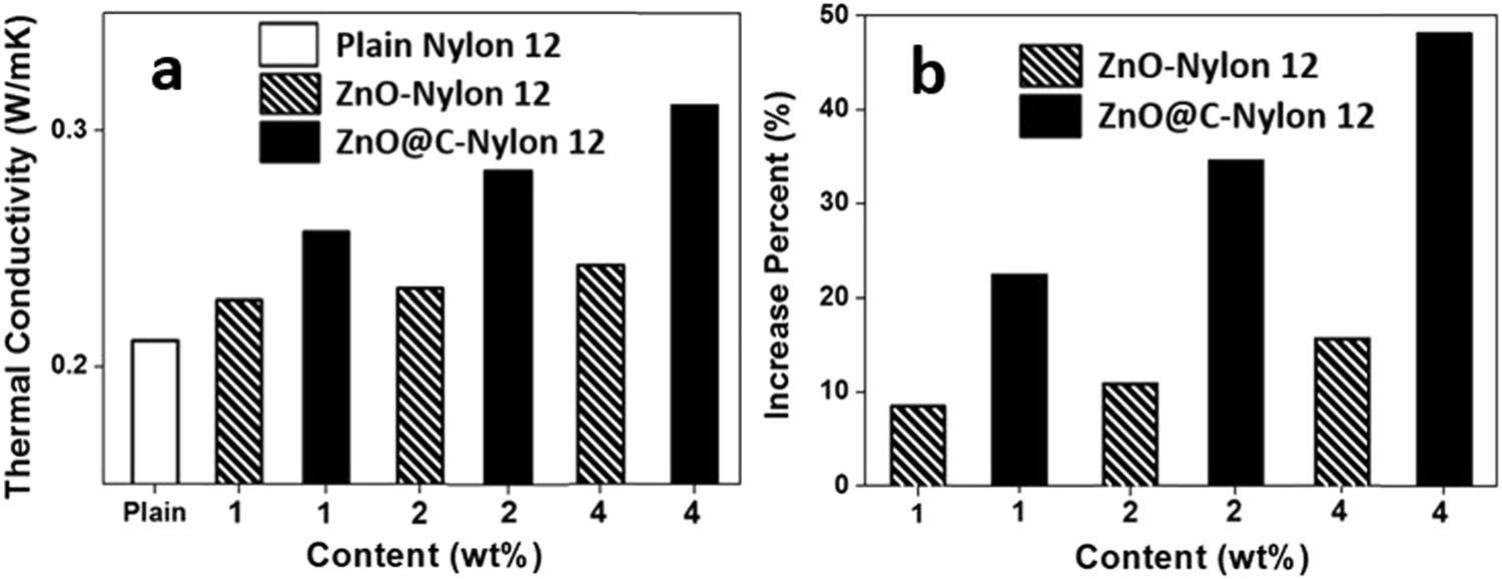
Thermal conductivity (**a**) and the % improvement (**b**) of the ZnO-nylon 12 and ZnO@C-nylon 12 nanocomposites. Measured at 25 °C.

The corresponding thermal conductivities of both composites, ZnO@C-nylon 12 and ZnO-nylon 12, are shown in Fig. 7. At 25 °C, the thermal conductivity of the neat nylon 12 is only 0.205 W∙m^−1^∙K^−1^, which sets the standard for comparison between the two composites. With the NP addition, the thermal conductivities have been improved for both composites, as shown in Fig. 7a, however at different enhancements for different amounts of NPs. These values increase gradually with increased NP contents, but the plain ZnO NPs-reinforced composites only show a marginal improvement whilst the ZnO@C NPs-reinforced composites exhibit a linear enhancement feature. The thermal conductivity of the ZnO@C NPs-reinforced samples is enhanced by 22, 35 and 49% (reaching an absolute value of 0.305 W∙m^−1^∙K^−1^) for the 1, 2 and 4 wt% NPs addition, respectively (Fig. 7b). At 4 wt% addition, the improvement of the ZnO@C -nylon 12 composite is 3 times higher than that of the neat ZnO-nylon 12. This result directly demonstrates the positive role of the C-coating in improving the thermal properties of a polymeric composite.

The level of enhancement reported here for the ZnO@C NPs is of significance, because the 49% improvement at 4 wt% of the core-shell NPs (where the core ZnO is much heavier than carbon) is virtually comparable to the effect of graphene as a filler to reinforce nylon composite^42^. Graphene has been reported to possess the best thermal conductivities (2000–4000 W∙m^−1^∙K^−1^)^41^. Therefore, the small amount of effective carbon in the NPs has generated a huge improvement, which indicates its high efficiency. This high efficiency is most likely originated from the improved dispersion and interface bonding of the ZnO@C NPs within the nylon 12 matrix. Detailed investigation will be followed as it is beyond the scope of this report.

## Conclusion

To sum up, we have demonstrated a simple, generalised, and multi-purpose RCVD system for the *in-situ* synthesis of uniform, controllable and highly graphitised C-coating on various metal oxides, to create nanoscale core-shell composite NPs. A vast amount of nano- and micro-sized particles can be effectively and nearly individually coated with an ultra-thin graphitic carbon layer of 1–5 nm thick, at large scale. The resulting core-shell composite NPs have indeed combined the advantages of graphitic carbon and metal oxides, benefiting their surface modification and dispersion in composite fabrication. Further investigation using ZnO@C NPs as an example filler in nylon 12 matrix has shown that the C-coating has led to a 49% improvement in thermal conductivity and 27% increase in tensile strength for the composites at a small amount of filler addition. This research opens up new opportunities for MO_X_@C core-shell NPs to be utilised in the creation of high performance polymeric nanocomposites with specifically desired functionalities based on the oxide cores.

## References

[1] Bystrzejewski M, Pyrzyńska K, Huczko A, Lange H (2009). Carbon-encapsulated magnetic nanoparticles as separable and mobile sorbents of heavy metal ions from aqueous solutions. Carbon.

[2] Kim J-K (2014). Effect of carbon coating methods on structural characteristics and electrochemical properties of carbon-coated lithium iron phosphate. Solid State Ionics.

[3] Zhang YJ (2014). Magnetron sputtering amorphous carbon coatings on metallic lithium: Towards promising anodes for lithium secondary batteries. J. Power. Sources..

[4] Ji L, Lin Z, Alcoutlabi M, Zhang X (2011). Recent developments in nanostructured anode materials for rechargeable lithium-ion batteries. Energ. Environ. Sci..

[5] Bhaviripudi S (2007). CVD synthesis of single-walled carbon nanotubes from gold nanoparticle catalysts. J. Am. Chem. Soc..

[6] Li Y, Zhou C, Xie X, Shi G, Qu L (2010). Spontaneous, catalyst-free formation of nitrogen-doped graphitic carbon nanocages. Carbon.

[7] Nie J, Qian W, Zhang Q, Wen Q, Wei F (2009). Very high-quality single-walled carbon nanotubes grown using a structured and tunable porous Fe/MgO catalyst. J. Phys. Chem. C..

[8] Jang J, Oh JH (2003). Facile fabrication of photochromic dye–conducting polymer core–shell nanomaterials and their photoluminescence. Adv. Mater..

[9] Petropoulos JP, Cristiani TR, Dongmo PB, Zide JMO (2011). A simple thermodynamic model for the doping and alloying of nanoparticles. Nanotechnology.

[10] Hung CM (2016). Synthesis and gas-sensing characteristics of α-Fe_2_O_3_ hollow balls. J. Science: Advanced Materials and Devices.

[11] Abbas N, Kim HT (2016). Multi-walled carbon nanotube/polyethersulfone nanocomposites for enhanced electrical conductivity, dielectric properties and efficient electromagnetic interference shielding at low thickness. Macromol. Res..

[12] Poizot P, Laruelle S, Grugeon S, Dupont L, Tarascon JM (2000). Nano-sized transition-metal oxides as negative-electrode materials for lithium-ion batteries. Nature.

[13] Van Thu L (2013). Surface modification and functionalization of carbon nanotube with some organic compounds. Adv. Nat. Sci:. Nanosci. Nanotechnol..

[14] Shafeeyan MS, Daud WMAW, Houshmand A, Shamiri A (2010). A review on surface modification of activated carbon for carbon dioxide adsorption. J. Anal. Appl. Pyrol..

[15] Ata MS, Liu Y, Zhitomirsky I (2014). A review of new methods of surface chemical modification, dispersion and electrophoretic deposition of metal oxide particles. RSC Adv..

[16] Dilsiz N (2000). Plasma surface modification of carbon fibers: a review. J. Adhes. Sci. Technol..

[17] Somani SP, Somani PR, Noda M, Umeno M (2008). Carbon nanocapsules encapsulating cobalt nanoparticles by pulsed discharge plasma chemical vapor deposition. Diam. Relat. Mater..

[18] Liu Y (2008). The confined growth of double-walled carbon nanotubes in porous catalysts by chemical vapor deposition. Carbon.

[19] Nakano H, Dokko K, Koizumi S, Tannai H, Kanamura K (2008). Hydrothermal synthesis of carbon-coated LiFePO_4_ and its application to lithium polymer battery. J. Electrochem. Soc..

[20] Zhang R, Hummelgård M, Olin H (2010). Carbon nanocages grown by gold templating. Carbon.

[21] Cui C (2013). Formation mechanism of carbon encapsulated Fe nanoparticles in the growth of single-/double-walled carbon nanotubes. Chem. Eng. J.

[22] Jourdain V, Bichara C (2013). Current understanding of the growth of carbon nanotubes in catalytic chemical vapour deposition. Carbon.

[23] Jin Z (2007). Ultralow feeding gas flow guiding growth of large-scale horizontally aligned single-walled carbon nanotube arrays. Nano Lett..

[24] White CM, Banks R, Hamerton I, Watts JF (2016). Characterisation of commercially CVD grown multi-walled carbon nanotubes for paint applications. Prog. Org. Coat..

[25] Liu J (2014). Multifunctional CoO@C metasequoia arrays for enhanced lithium storage. Nano Energy.

[26] Xu F, Wang N, Chang H, Xia Y, Zhu Y (2014). Continuous production of IF-WS_2_ nanoparticles by a rotary process. Inorganics.

[27] Spitalsky Z, Tasis D, Papagelis K, Galiotis C (2010). Carbon nanotube–polymer composites: chemistry, processing, mechanical and electrical properties. Prog. Polym. Sci..

[28] Chatterjee S (2012). Size and synergy effects of nanofiller hybrids including graphene nanoplatelets and carbon nanotubes in mechanical properties of epoxy composites. Carbon.

[29] Xu, F. *et al*. Multi-walled carbon/IF-WS_2_ nanoparticles with improved thermal properties. *Nanoscale* **5** (2013). 10.1039/c3nr03844k 24057128

[30] Xu F (2014). Ultra-toughened nylon 12 nanocomposites reinforced with IF-WS_2_. Nanotechnology.

[31] Yang Z-C (2013). Cobalt monoxide-doped porous graphitic carbon microspheres for supercapacitor application. Sci. Rep-UK.

[32] Sathasivam S (2015). Tungsten Doped TiO2 with Enhanced Photocatalytic and Optoelectrical Properties via Aerosol Assisted Chemical Vapor Deposition. Sci. Rep-UK.

[33] Ferrari AC (2007). Raman spectroscopy of graphene and graphite: Disorder, electron–phonon coupling, doping and nonadiabatic effects. Solid State Commun..

[34] Malard LM, Pimenta MA, Dresselhaus G, Dresselhaus MS (2009). Raman spectroscopy in graphene. Phys. Rep..

[35] Zheng M (2010). Metal-catalyzed crystallization of amorphous carbon to graphene. Appl. Phys. Lett..

[36] Celorrio V (2016). Influence of thermal treatments on the stability of Pd nanoparticles supported on graphitised ordered mesoporous carbons. Int. J. Hydrogen Energ..

[37] Nine MJ, Cole MA, Tran DNH, Losic D (2015). Graphene: a multipurpose material for protective coatings. J. Mater. Chem. A.

[38] Sun Z (2007). Coating carbon nanotubes with metal oxides in a supercritical carbon dioxide–ethanol solution. Carbon.

[39] Hee BK, Kiyoshi N, Sung HL, Daisuke S (1998). Epitaxial Growth of ZnO Films on (0001) Sapphire at Low Temperatures by Electron Cyclotron Resonance-assisted Molecular Beam Epitaxy and Their Microstructural Characterizations. Jpn. J. Appl. Phys..

[40] Kaseem M, Hamad K, Ko YG (2016). Fabrication and materials properties of polystyrene/carbon nanotube (PS/CNT) composites: A review. Eur. Polym. J..

[41] Kalin M, Zalaznik M, Novak S (2015). Wear and friction behaviour of poly-ether-ether-ketone (PEEK) filled with graphene, WS_2_ and CNT nanoparticles. Wear.

[42] Ding P (2014). Highly thermal conductive composites with polyamide-6 covalently-grafted graphene by an *in situ* polymerization and thermal reduction process. Carbon.

